# Periapical Actinomycosis: A Rare Subdivision of Cervicofacial Actinomycosis, Review of the Literature, and a Case Report

**DOI:** 10.1155/2022/7323268

**Published:** 2022-06-06

**Authors:** Ramtin Dastgir, Maryam Sohrabi

**Affiliations:** ^1^Faculty of Dentistry, Tehran Medical Sciences, Islamic Azad University, Tehran, Iran; ^2^Department of Oral and Maxillofacial Surgery, School of Dentistry, Tehran University of Medical Sciences, Tehran, Iran

## Abstract

**Background:**

Periapical actinomycosis, which is reckoned as a subgroup of cervicofacial actinomycosis, is an uncommon, more indolent, less invasive, and limited type of actinomycosis infection. However, it can be considerably underreported due to the low number of periapical surgical specimens that are submitted for histopathological analysis after excision of the lesion with the preliminary diagnosis of typical periapical infections. It is believed that during root canal treatment, the organisms are displaced from the oral cavity into the periapical regions as a result of failure to establish aseptic techniques which can further result in actinomycosis infections and, in rare instances, lead to more severe events and can even be life-threatening. *Case Presentation*. We intend to report a case of periapical actinomycosis in a 34-year-old female who presented with the chief complaint of pain and slight mobility of the mandibular right second premolar and first molar with no significant issues in the patient's medical history. Initial orthopantomography revealed a uniloculated, radiolucent lesion engulfing the apices of the aforementioned teeth. An incisional biopsy was then obtained which revealed fragments of fibroconnective tissue including few crushed bone particles severely infiltrated by acute inflammatory cells and some foamy macrophages. The suppurative exudate focally surrounds colonies of filamentous bacteria as round basophilic masses with radial configuration resembling “sulfur granules.” Surgical approach consisted of curettage accompanied with peripheral ostectomy and cautious burnishing of the two involved tooth roots.

**Conclusion:**

This case report emphasizes the importance of aseptic techniques during endodontic and more invasive treatments, as they can cause penetration of Actinomyces into the periapical region which in some cases can lead to more serious complications and even life-threatening situations.

## 1. Introduction

Actinomycosis, often called the “great imitator”, is a rare chronic granulomatous infection caused by Gram-positive, non-acid-fast, branched filamentous, anaerobic, or microaerophilic/capnophilic bacillus bacteria of the *Actinomyces* genus. The *Actinomyces* genus belongs to family *Actinomycetaceae* (the single member of order *Actinomycetales*) that also includes *Arcanobacterium*, *Actinobaculum*, *Mobiluncus*, *Trueperella*, and *Varibaculum*. They mainly colonize the oropharynx, gastrointestinal tract, and female urogenital tract [1–4]. Furthermore, *Actinomyces* spp. are normal commensals of the human oropharynx, gastrointestinal tract, and genitourinary tract [4]. Actinomycosis infection is most commonly observed in the cervicofacial region and accounts for almost 55% of all actinomycosis infection cases [5]. This infection is most commonly due to *Actinomyces israelii* or less commonly other etiologic species, such as *A. odontolyticus*, *A. naeslundii*, *A. viscosus*, *A. propionica*, *Propionibacterium propionicus*, and *A. gerencseriae* [6, 7]. The reason behind the notorious nickname of the “great imitator” can perhaps be attributed to the challenging diagnosis of this infection due to its nonspecific radiological findings and nonspecific symptoms. Despite occurring rarely, actinomycosis should be included in the differential diagnosis when encountered with a soft-tissue mass with inflammatory changes with an infiltrative nature in the cervicofacial area in radiographic images. Moreover, it should be noted that CT scan and MRI are not sufficient for distinguishing actinomycosis from malignant tumoral masses [8]. Actinomycosis infection is characterized by suppurative and granulomatous lesions which primarily occur in soft tissues. Though rare, primary cutaneous and skeletal involvements are also probable [9–11]. Main risk factors which leave the patient vulnerable to actinomycosis infection include mucosal breach, impaired local or systemic immune defenses, poor oral hygiene, facial trauma, prior history of head and neck radiation, or oral surgeries. However, few cases of actinomycosis infection have been described where the etiology of infection could not be accurately specified [1, 5, 8]. Furthermore, this infection more commonly occurs in rural areas than urban areas [8].

We intend to report an unusual case of periapical actinomycosis which presented to our oral and maxillofacial surgery clinic with a chief complaint of pain in the posterior mandible region and further review the available literature.

## 2. Case Presentation

A 34-year-old female patient presented to our oral and maxillofacial surgery private practice office with the chief complaint of pain and slight mobility of the mandibular right second premolar and first molar. There were no significant issues in the patient's medical history upon presentation. The patient signed a written consent stating her approval for participation in this report and the publication of her clinical findings, photographs, radiographs, histopathologic findings, and results to be reported in this article.

Clinical examination revealed pain on percussion on the mandibular second premolar and first molar. Slight mobility of these two teeth was also evident on palpation. Overlying skin and mucosa of the region was normal and devoid of any ulcerations, erythema, suppurations, and rise in temperature. Intraoral examination did not reveal any breach in the mucosa and the integrity of it remained intact. On palpation of the oral mucosa, the consistency was bony hard on both lingual and buccal cortices.

**Figure 1:**
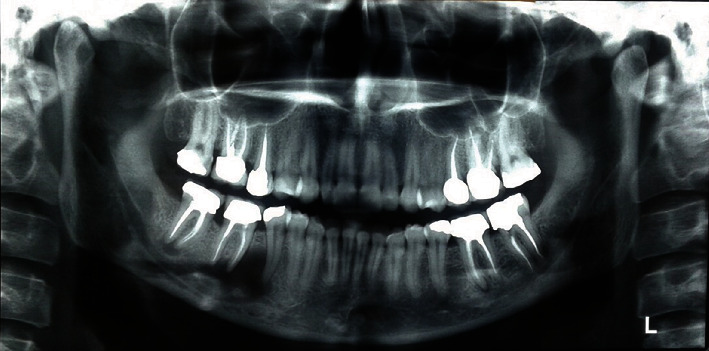
Initial OPG of the patient depicting a unilocular radiolucency encompassing the apices of the mandibular second premolar and first molar.

**Figure 2:**
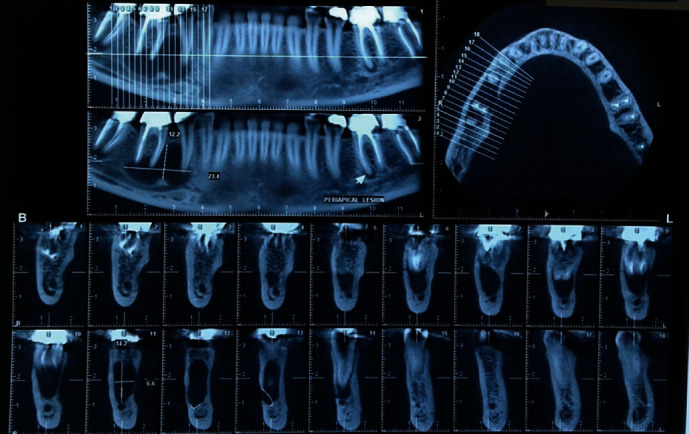
Initial CBCT obtained from the patient further revealing the extents of the lesion.

An initial orthopantomography (OPG) was obtained which revealed a uniloculated, radiolucent lesion engulfing the apices of the mandibular right second premolar and an endodontically treated first molar, extending to the superior border of the inferior alveolar nerve canal with an irregular shape dissimilar to what is routinely observed in periapical cysts or granulomas associated with endodontically treated teeth (Figure 1). The patient claimed that the mandibular right first molar was endodontically treated almost 5 years prior to presentation. Subsequently, a cone beam computed tomography (CBCT) was ordered which further revealed the extensions of the lesion in the buccolingual aspect, almost completely obliterating the lingual cortex. The lesion measured 23.4 × 14.2 × 6.6 mm at its peaks (Figure 2). Preliminary differential diagnosis based on the radiographic evaluation included odontogenic keratocyst (OKC), radicular cyst, and unicystic ameloblastoma.

**Figure 3:**
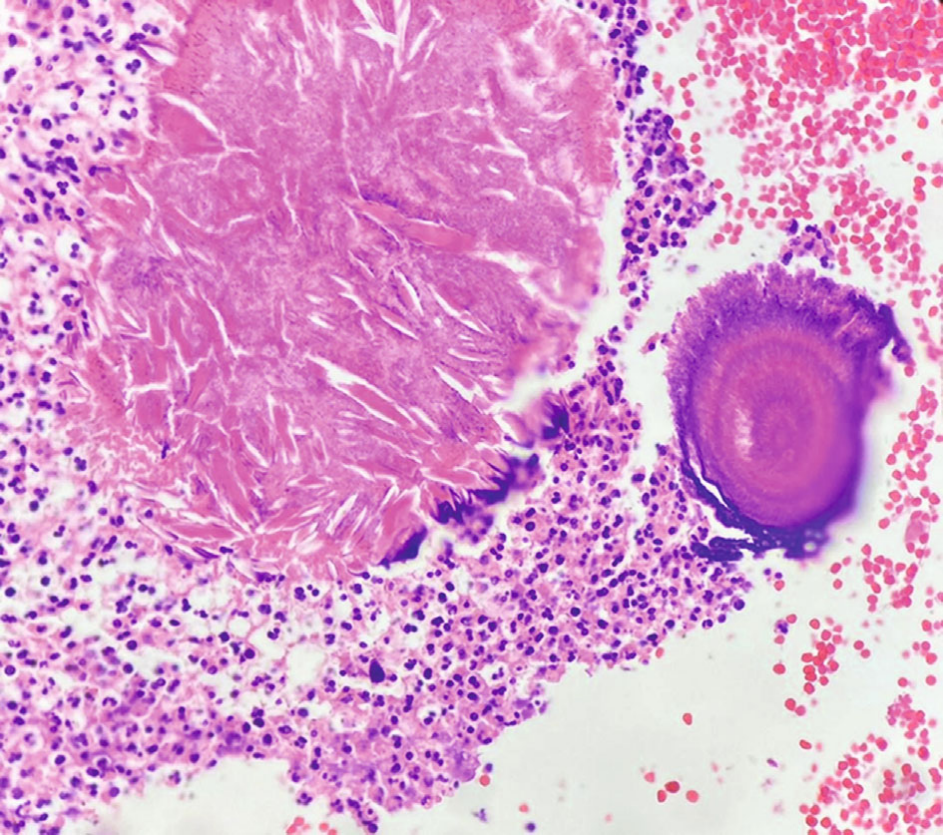
Microscopic findings of fibroconnective tissue containing few crushed bone particles severely infiltrated by acute inflammatory cells and some foamy macrophages (H&E staining, magnification level 10 × 40).

**Figure 4:**
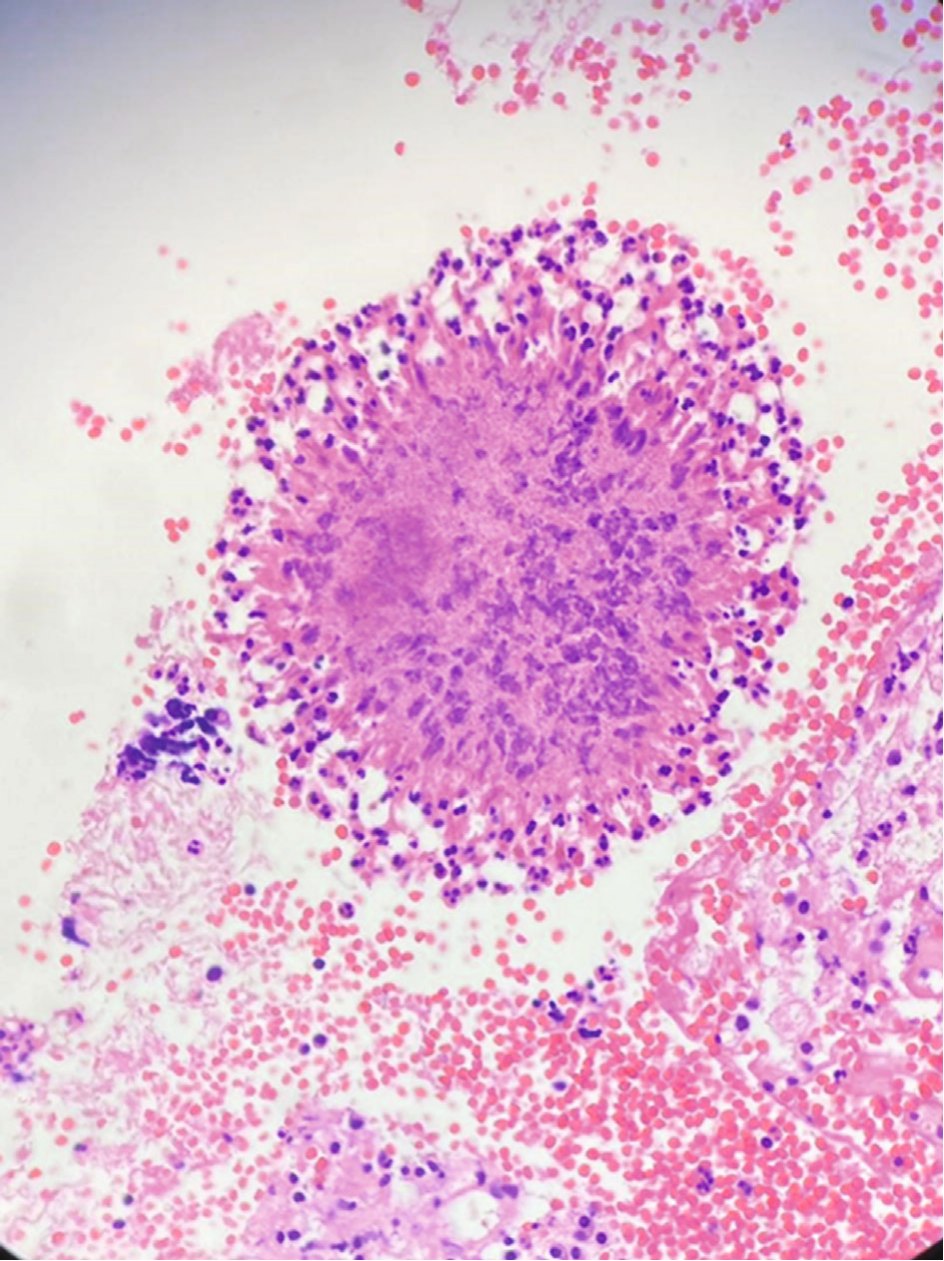
The suppurative exudate focally surrounding colonies of filamentous bacteria as round basophilic masses with radial configuration resembling “sulfur granules” (H&E staining, magnification level 10 × 40).

Following these observations and suspicion towards something more malignant, an incisional biopsy was obtained. The incised specimen consisted of multiple fragmented and irregular pieces of soft, creamy-colored tissue. Microscopic evaluations revealed fragments of fibroconnective tissue including few crushed bone particles severely infiltrated by acute inflammatory cells and some foamy macrophages (Figure 3). The suppurative exudate focally surrounds colonies of filamentous bacteria as round basophilic masses with radial configuration resembling “sulfur granules” (Figure 4). Following the consistency between the microscopic findings of our case and of actinomycosis, the definitive diagnosis of periapical actinomycosis was made.

**Figure 5:**
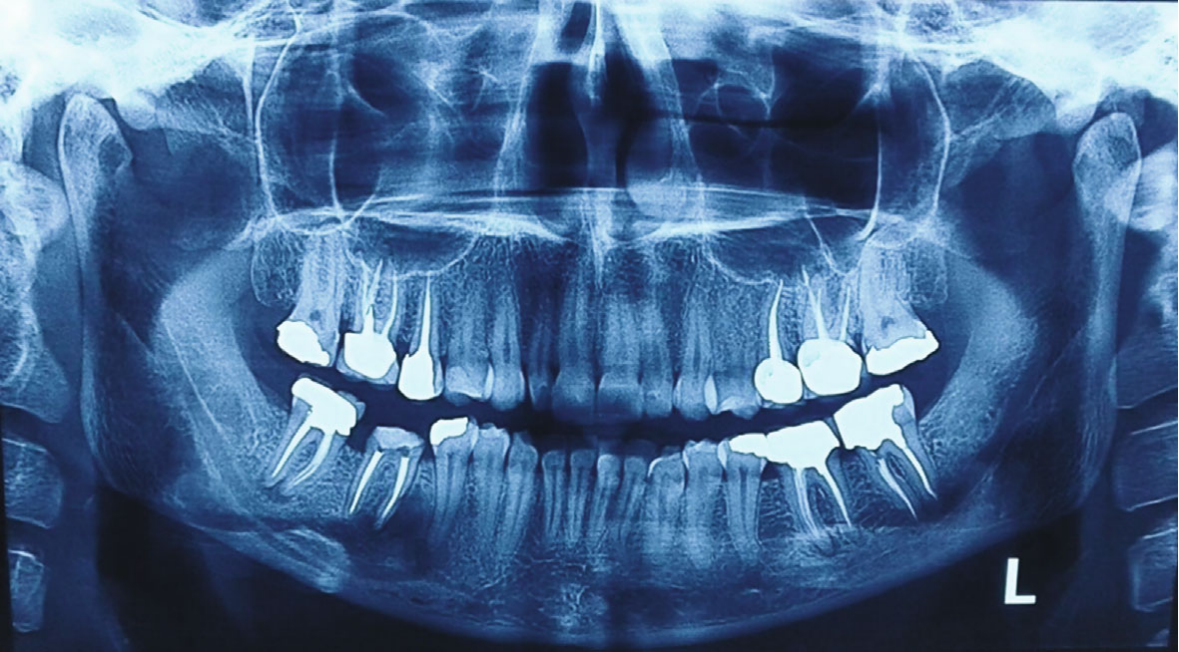
Mandibular second molar was endodontically retreated and referred back to us for further evaluation.

Upon the diagnosis, the patient was referred to an infectious diseases specialist and an endodontist for further consultations. Antibiotics were not prescribed by the infectious diseases specialist due to the limited and contained nature of the lesion. However, the endodontist endodontically retreated the tooth and the patient was referred back to us for the subsequent periapical surgery (Figure 5). After obtaining a written informed consent form from the patient, the surgery was performed under local anesthesia. Surgical procedure incorporated inferior alveolar nerve block accompanied with mental nerve block, followed by a full-thickness periosteal flap elevation with great vigilance in exposing and preserving the mental nerve and subsequent curettage accompanied with peripheral ostectomy and cautious burnishing of the two involved teeth roots. Subsequently, the flap was reapproximated and sutured in place. Furthermore, one-year follow-up of the patient did not show any evidence of recurrence.

## 3. Discussion and Conclusion

Actinomycosis is a rare, chronic, acute or subacute, invasive bacterial infection with an annual incidence rate of approximately 0.00003% [12, 13]. Bacteria of the genus *Actinomyces* belong to the Actinobacteria phylum and Actinomycetales order and are related to other genera such as *Corynebacterium*, *Mycobacterium*, *Nocardia*, and *Propionibacterium*. More than 30 species of *Actinomyces* have been identified. *Actinomyces israelii* is the most prevalent species isolated in human infections and is found in most clinical forms of actinomycosis infections [1, 13–18]. *A. israelii* and *A. gerencseriae* are responsible for almost 70% of actinomycotic orocervicofacial infections [17]. It should be noted that most of the *Actinomyces* spp. are present in polymicrobial flora. Hence, *Actinomyces* are often isolated with other normal commensals, such as *Aggregatibacter actinomycetemcomitans*, *Capnocytophaga*, *Eikenella corrodens*, fusobacteria, *Bacteroides*, streptococci, staphylococci, or Enterobacteriaceae, depending on the site of infection [16].

Cervicofacial actinomycosis is the most frequently observed clinical form of actinomycosis infections. Furthermore, “lumpy jaw syndrome” which is associated with odontogenic infections is its most common clinical manifestation which constitutes approximately 60% of all reported cases of actinomycosis and shows a male predominance of 1.5-3.1 times [1, 10, 15, 19]. *A. israelii* and *A. gerencseriae* are observed in almost 70% of cervicofacial actinomycosis cases; however, other species have also been described in such infections, including *A. meyeri*, *A. odontolyticus*, *A. naeslundii*, *Actinomyces georgiae*, *Actinomyces pyogenes*, or *A. viscosus* [17]. *Actinomyces* are also commensals of the human oropharynx, and their prevalence is particularly noted within dental plaques, gingival crevices, tonsillar crypts, and periodontal pockets and on carious teeth [1, 14, 15]. Therefore, dental procedures such as dental extractions and root canal therapy and underlying oral conditions which predispose the oral health to vulnerability such as caries, periodontal diseases, and poor oral hygiene seem to play a crucial role in its occurrence [10, 20]. Cervicofacial actinomycosis could be associated with large abscesses and/or osteomyelitis with or without sinus tract. Furthermore, cervicofacial actinomycosis can lead to invasion of the adjacent vital structures such as skull base as well as distant organ dissemination, such as the brain, lungs, and gastrointestinal tract [21, 22].

Cervicofacial actinomycosis can be further divided into central and peripheral subtypes, of which the central subtype is considerably uncommon with the incidence of just 1–2%. Periapical actinomycosis is categorized under central subtype which itself is very rare among central varieties [13, 23, 24]. Periapical actinomycosis, which is reckoned as a central subgroup of cervicofacial actinomycosis, is an uncommon, more indolent, less invasive, and limited type of infection that has been reported in less than just 5% of periapical lesions [25–28]. However, it can be considerably underreported due to the low number of periapical surgical specimens that are submitted for histopathological analysis after excision of the lesion with the preliminary diagnosis of typical periapical infections [20]. Most cases of periapical actinomycosis have been reported individually, and data regarding the prevalence of periapical actinomycosis among periapical lesions is limited to only a few studies [25, 27, 28]. However, there is dearth of literature supporting the mode of entry of actinomycosis into the periapical region. Nevertheless, it is believed that during root canal treatment, the organisms are displaced from the oral cavity into the periapical regions as a result of failure to establish aseptic techniques. Since the majority of actinomycosis cases described in the literature have been reported in endodontically treated teeth, it can further cement the aforementioned hypothesis [24, 25, 29]. Penetration of the bacteria through the impaired periodontium has also been hypothesized to play an important role in periapical actinomycosis [30].

Radiologically, periapical actinomycosis usually presents as well-defined radiolucent lesion located in the apical region of an endodontically treated maxillary or mandibular teeth which does not entail a pivotal diagnostic value as it can be misinterpreted with a variety of odontogenic lesions, most specifically a periapical granuloma or abscess [25]. As seen in our case, it presented as a uniloculated, radiolucent lesion encompassing apices of the right mandibular first molar and second premolar.

Histopathologically, microscopic findings for actinomycosis indicate two zones: an outer zone of granulation and a central zone with multiple granules representing colonies of Actinomyces. Histopathological analysis discloses one to three sulfur granules in about 75% of cases, described as basophilic masses with eosinophilic terminal clubs on staining with hematoxylin and eosin. Classic microscopic findings include necrosis with yellowish sulfur granules and filamentous Gram-positive fungal-like pathogens. Yellow sulfur granules are established by aggregation of bacteria trapped in biofilm [31–33]. However, sulfur granules are not pathognomonic, as they are only present in 35–55% of actinomycosis cases and are also seen with *Nocardia*, *Streptomyces*, and *Peptostreptococcus* [3, 9, 23]. Tissue culture is the most precise method of diagnosis of actinomycosis. However, it is limited in practice since *Actinomyces* species have difficulty growing due to their anaerobic nature [9]. More recently, molecular and genetic techniques involving immunofluorescence, fluorescent in situ hybridization, and 16S rRNA analysis with polymerase chain reaction (PCR) have been utilized to aid diagnosis of actinomycosis [15, 34–37].

Treatment of cervicofacial actinomycosis primarily consists of surgery of incision and drainage in conjunction with long-term antibiotic therapy. *Actinomyces* spp. are usually extremely susceptible to beta-lactams, especially penicillin G or amoxicillin. As a result, penicillin G or amoxicillin is considered a drug of choice for the treatment of actinomycosis. It should be noted that inadequate short-term antibiotic therapy can result in the relapse of infection. Furthermore, drug resistance is not an issue in treatment of actinomycosis [2, 14, 15, 38]. Treatment of periapical actinomycosis however differs from the treatment of other cervicofacial actinomycosis infections. Endodontic treatment or retreatment is usually recommended for elimination of the intrapulpal source of infection, with or without periradicular surgery when an extraradicular infection is believed to have established a periapical lesion. Furthermore, dental extraction can also be of value in elimination of the local infection. Therefore, antibiotic therapy may be of limited value, with regard to evidences that it cannot sufficiently reach the infected necrotic periapical regions at adequate concentrations [13, 39]. For this same reason, the infectious diseases specialist we consulted did not prescribe any antibiotic regimen for our patient and insisted on avoiding overusing antibiotics. In a recent study, Butera et al. evaluated the efficacy of probiotics/paraprobiotics in periodontal diseases. In their study, it was demonstrated that hyaluronic acid and lactoferrin appear as reliable approaches for the management of periodontal disease. Furthermore, paraprobiotics are likely to demonstrate the most important benefit due to their immunomodulating mechanism of action [40]. The results of this study can be considered for future evaluation on the role of probiotics/paraprobiotics for actinomycosis infections as well as different oral infections mainly due to their immunomodulation effects.

Periapical actinomycosis is a very rare subtype of cervicofacial actinomycosis that presents with nonspecific clinical and radiological manifestations. Though not totally accurate, conventional biopsies and hematoxylin and eosin (H&E) staining in addition to histochemical stains and in conjunction with clinical signs and symptoms can be beneficial in reaching a definitive diagnosis. The current evidence is not adequate to determine a definitive treatment for periapical actinomycosis; however, surgical resection of the lesion seems to be the treatment of choice as antibiotic treatment seems to be unnecessary in such cases.

Our case report further emphasizes the importance of aseptic techniques during endodontic and more invasive treatments, as they can cause penetration of Actinomyces into the periapical region which in some cases can lead to more serious complications and even life-threatening situations.

## Data Availability

Data sharing is not applicable to this article as no datasets were generated or analysed during the current study.

## Abbreviations

A. odontolyticus: Actinomyces odontolyticus
A. naeslundii: Actinomyces naeslundii
A. viscosus: Actinomyces viscosus
A. propionica: Actinomyces propionica
A. gerencseriae: Actinomyces gerencseriae
A. meyeri: Actinomyces meyeri
OPG: Orthopantomography
CBCT: Cone beam computed tomography
OKC: Odontogenic keratocyst
spp.: Species
rRNA: Ribosomal RNA
PCR: Polymerase chain reaction
H&E: Hematoxylin and eosin.
